# Strengthening environmental health services delivery through improving data management in South Africa: insights from environmental health managers

**DOI:** 10.3389/frhs.2025.1665259

**Published:** 2025-11-06

**Authors:** Siphesihle Siyamukela Masimula, Mpinane Flory Senekane, Nisha Naicker

**Affiliations:** 1 Department of Environmental Health, Faculty of Health Sciences, University of Johannesburg, Johannesburg, South Africa; 2 National Institute for Occupational Health, National Health Laboratory Services, Johannesburg, South Africa

**Keywords:** data use, environmental health data management, environmental health information system, environmental health services, municipalities, public health

## Abstract

**Background:**

In the delivery of environmental health services (EHS), the data that gets collected provides an opportunity to depict the extent of environmental threats to human health in communities and inform required interventions. In this study, the perspectives and role of environmental health managers in improving data management in the delivery of EHS in municipalities in the KwaZulu-Natal province of South Africa were assessed.

**Methods:**

A qualitative phenomenological approach was followed. Data were collected from ten managers via a semi-structured interview guide from February 2024 to April 2024. The transcripts derived from the interviews were analysed via ATLAS.ti version 24.0.0.29576, following which deductive and inductive thematic analysis methods were used.

**Results:**

The results revealed various roles and responsibilities that environmental health managers play to improve data management and enable insight-driven decision-making. Furthermore, it was shown that through data-driven insights, EHS delivery can be strengthened to be impactful and enable better health outcomes in communities amid existing institutional and technical challenges. This indicated a need for a holistic approach to review and modernise environmental health data management in South Africa to maximise available opportunities.

**Conclusion:**

In this juncture, managers have a duty to drive transformation, apply change management and instil a culture of data use in their institutions for impactful service delivery.

## 1 Introduction

Environmental health services (EHS) are functions that are provided by the government to assess, monitor, correct, prevent, and control environmental factors that can adversely affect human health (1). These factors include, inter alia, air quality, water quality, food safety and hygiene, the management of waste, climate change, occupational health and safety, the proliferation of vectors, communicable diseases and hazardous substances (2, 3). The delivery of EHS, in different parts of the world, is provided through countries' public health systems as a key part of a preventative and health-promoting healthcare approach (4). In South Africa, the majority of EHS are provided at the municipal level, whereby environmental health practitioners (EHPs) and other allied professionals are employed to play a significant role in ensuring that environments where communities live, learn, work, and play are healthy and safe for their well-being (5). In the delivery of these services, the management and utilisation of the data that are collected are central to informing planning and decision-making through depicting the extent of environmental health risks and guiding the interventions required to protect people's health (6). When integrated with other clinical datasets, environmental health data provide an excellent opportunity for different health services to collaborate and join efforts to address cross-cutting issues and better understand the link between the state of the environment and the burden of disease (7). This shows the importance of continuously strengthening environmental health data management and utilisation. In order to improve environmental health surveillance, risk communication, community engagement, research and innovation, as well as enabling multisectoral collaborations for sustainable communities and better health outcomes.

In South Africa, environmental health data are collected and managed mainly at a service-rendering level in municipalities that provide EHS (8). This is where EHPs collect and report data on environmental health indicators to the country's District Health Information System (DHIS), which is the country's broader routine health information system for almost all health services. These environmental health indicators within the DHIS, constitute the country's environmental health information system. In this data management process, environmental health managers are responsible for data verification, consolidation and timeous submission to the next level of the health system until the data reaches the national level (8). In this process, inter-alia, environmental health managers are entrusted with other roles, including supervising EHPs, managing working resources, keeping records and managing collected data, ensuring data quality, and using data to inform the delivery of EHS and strategic planning in their settings. As a result, it is very important for managers in the delivery of health services to understand the potential value of the data they generate and the information that is at their disposal (9). The collection of data goes beyond meeting reporting requirements or demands. Hence, managers, as organisational leaders, must provide strategic leadership in ensuring that collected routine health data and its utilisation align with key performance indicators and broader institutional business strategies in the provision of health services (10). Skiti (11) postulates that strategic leadership, managerial support, motivation and culture-driven data ownership have a direct effect on the processes and performance of routine health information systems. This places managers at a strategic point in the data flow process to ensure that data management and its utilisation are optimal to enhance service delivery.

In this study, the perspectives of environmental health managers on data management in the delivery of EHS in KwaZulu-Natal municipalities in South Africa were assessed, using a qualitative approach. This assessment highlights the role of environmental health managers in data management and their perceptions and challenges as key players in the country's environmental health information system. This was important for identifying areas in need of improvement and best practices to be shared and optimised to strengthen the delivery of EHS through improved data management processes and utilisation from the managers' point of view. This study was conducted as part of a broader research project that evaluated environmental health data management in municipalities in the province of KwaZulu-Natal, South Africa.

## 2 Materials and methods

### 2.1 Study design and setting

A phenomenological qualitative approach was employed in this study to explore environmental health managers' perspectives relative to environmental health data management. This qualitative research strategy seeks to describe the essence of a phenomenon by exploring and understanding it from the perspective of those with lived experiences (12, 13). This qualitative approach was used to gain a better understanding of environmental health data management in the provision of EHS in municipalities from multiple perspectives. As a result, this study was conducted in the municipalities that provide EHS in the province of KwaZulu-Natal in South Africa due to their accessibility to the researchers, and in response to environmental health issues requiring a data science approach. The KwaZulu-Natal Province is the second most populated administrative region of South Africa, with a population of about 12.4 million (14). Similar, to the rest of South Africa, this province faces environmental health challenges that include climate change related disasters, emerging and re-emerging infectious diseases, rapid increase of vectors, poor waste management, growth of informal settlements, water scarcity and quality issues, as well as sporadic foodborne illnesses and chemical poisoning outbreaks (15–20). These challenges affect communities at a local level of municipalities. This raises the importance of strengthened EHS and localised interventions, through improved data management and utilisation. As part of the unitary state of South Africa, the KwaZulu-Natal province reports environmental health data to the DHIS through 13 EHS rendering municipalities (8, 21). Therefore, for this study to be conducted, 13 municipalities were approached and requested to form part of the study, and permission was sought in 11 municipalities.

### 2.2 Participants selection

In the 11 municipalities, 42 environmental health managers were eligible to form part of the study. They were involved in the management of environmental health data and registered with the Health Professions Council of South Africa under the Environmental Health Board as independent EHPs. From a population of 42 managers, 16 managers were identified and selected for recruitment to form part of the study, as shown in Table 1. During the identification and selection for recruitment of the 16 managers, the representatives of the municipalities were asked to provide lists of all environmental health managers in their respective municipalities and indicate their responsibilities. The researchers purposively selected the 16 managers from those lists based on their occupational responsibilities and hands on experience of more than 10 years in relation to the research phenomenon. It was considered that since these managers are trusted with these responsibilities, they have knowledge and expertise in the provision of EHS and the management of environmental health data; therefore, their contribution to this study would be beneficial. Hence, they were purposively selected to obtain detailed information on the subject matter because of their key involvement in the management and utilisation of environmental health data in their respective municipalities. Subsequently, 16 managers from the 11 municipalities were chosen to ensure adequate representation. More than 11 managers were purposively selected because some municipalities had managers responsible for the broader management of EHS and those who were assigned with specific auxiliary responsibilities (i.e., monitoring and evaluation, quality assurance, and environmental health information system administration) that closely related to this study. Hence, in those municipalities (*n* = 4), more than one manager was selected for recruitment. Their participation and contribution were deemed impactful in providing data to meet the study's research objectives. Among the 16 managers who were recruited, 10 responded positively and agreed to participate in the study by signing an informed consent form, yielding a response rate of 62.5%. Table 1 also shows the distribution of the managers who agreed to participate. Owing to the confidential nature of this study, the names of the municipalities were replaced with different identification codes.

**Table 1:** Distribution of the recruitment and participation of environmental health managers.

Municipalities in codes	Total number of managers per municipality	Number of managers requested to participate	Number of managers who participated
#001	2	2	1
#002	1	1	0
#003	1	1	1
#004	1	1	0
#005	1	1	0
#006	1	1	0
#007	3	1	1
#008	19	4	4
#009	4	2	1
#010	4	1	1
#011	5	2	1
Total	42	16	10

### 2.3 Data collection

The data for this study were collected from the 1st of February 2024 to the end of April 2024 through interviews with environmental health managers. A semi-structured interview guide was used as a research instrument to guide the collection of qualitative data in relation to the phenomenon and promote consistency. The interview guide comprised predetermined closed-ended demographic questions and open-ended questions on roles and responsibilities, quality assurance activities, use of data for planning and decision-making, experienced challenges and prospective solutions to strengthen EHS through improved data management practices and systems. Ten managers who agreed to form part of the study were interviewed once, and data saturation was reached, with the information they provided. Individual interviews were conducted by the first author in a manner and setting that suited the preferences of the participants in terms of their availability, mode and comfort. Four interviews were conducted in person (face-to-face), and the other six were conducted virtually. The participants were encouraged to talk freely and were able to share their perceptions, experiences, knowledge, challenges and recommendations on the research subject, as their participation was confidential. In all the interviews, the first author manually transcribed the data and kept the transcripts for data analysis.

### 2.4 Data analysis

Ten transcripts derived from the interviews were imported into ATLAS.ti version 24.0.0.29576, whereby the deductive and inductive thematic analysis methods were applied to analyse the data. The use of this software enabled smooth organisation of data for reading, familiarisation and interactive visualisation for systematic analysis and interpretation. Following the deductive thematic analysis method, the data in the transcripts were segmented into quotations that reflected different statements by the participants. These quotations were then deductively coded according to the predetermined themes outlined in Table 2 to enable further analysis. These coded quotations were then read for analysis, interpreted in relation to the phenomenon of interest in this study and used to generate results. To further analyse the imported data, inductive thematic analysis was also conducted to observe patterns from the data and determine new emerging themes of interest in relation to the study to generate more results. However, no new codes emerged from the data, in line with the purpose of this study. As a result, code saturation was reached through the predetermined themes.

**Table 2:** Predetermined themes for deductive coding in the analysis of qualitative data from the interviews.

Code number	Predetermined themes
C0	Interviewees’ demographic information.
C1	Managers’ roles and responsibilities in relation to environmental health data management.
C2	Activities that managers engage in to ensure data quality in their areas of responsibility.
C4	Use of environmental health data to advance planning and decision-making.
C5	Challenges in environmental health data and information management.
C6	Prospective solutions to improve environmental health data management and utilisation.

### 2.5 Trustworthiness

To ensure trustworthiness in this study, attention was given to credibility, dependability, transferability and confirmability, as described by Polit and Beck (22). Credibility was promoted by persistently reading the qualitative data obtained from the interviews to ensure proper interpretation and reflection of the participants' responses. To enhance dependability, interviews and transcription were conducted by the same researcher (first author), guided by a standard semi-structured interview guide. A consistent approach was used for analysing all the transcripts from the interviews. Transferability was established through describing the data collection and analysis methods. For confirmability and to eliminate researcher bias, all stages of data analysis were documented to create an audit trail, demonstrating the rationale used in the development of the research findings. The second and third authors provided oversight on the methodology and data analysis process. Quotations are used in the presentation of the results.

### 2.6 Ethics approval

Ethical approval (REC-2469-2023) for this study was received from the Faculty of Health Sciences Research Ethics Committee at the University of Johannesburg. The permission to conduct this study was granted by the South African Local Government Association, and the management of the municipalities that participated. Informed consent was obtained from all the participants for their voluntary participation. Participants were informed of their right to withdraw from the study at any time. Participants were also treated with respect, fairness and human dignity. No harm to participants took place. Privacy and confidentiality were ensured.

## 3 Results

### 3.1 Demographics and characteristics of the study

Baseline demographics and characteristics of the study are reported in Table 3. The participants included eight males and two females aged 38–59 years. All the managers held postgraduate qualifications, in addition to their undergraduate Environmental Health qualifications (Diploma), and they had vast experience in environmental health. In terms of assigned roles in their respective municipalities, seven managers reported coordinating the provision of EHS and supervising EHPs. Three were responsible for quality assurance, monitoring and evaluation, and the administration of environmental health data repositories and systems.

**Table 3:** Demographic characteristics of the participants.

Participant	Gender	Age	Highest educational qualification	Years of experience	Areas of responsibility
1	Female	53	Bachelor's Degree in Management	30 years as an EHP and 3 years as a manager	Coordination of the delivery of EHS
2	Male	54	Bachelor of Technology in Environmental Health	11 years as a manager	Coordination of the delivery of EHS
3	Female	52	Postgraduate Diploma in Occupational Health	26 years in Environmental Health	Coordination of the delivery of EHS
4	Male	38	Postgraduate Diploma in Public Administration	15 years in Environmental Health	Coordination of the delivery of EHS
5	Male	58	Master's Degree in Chemical Engineering	23 years in Environmental Health	Quality assurance; Monitoring and evaluation
6	Male	55	Bachelor of Technology in Environmental Health	33 years in Environmental Health	Coordination of the delivery of EHS
7	Male	40	Bachelor of Technology in Environmental Health	17 years in Environmental Health	Coordination of the delivery of EHS
8	Male	47	Bachelor of Technology Degree in Environmental Health	10 years at an operational level, and 14 years at a management level	Quality assurance; Administration of internal environmental health data repositories
9	Male	45	Master of Medical Sciences in Public Health	24 years in Environmental Health and Public Health, of which 17 years are at a management level.	Administration of internal environmental health data repositories and information processing
10	Male	52	Master of Business Administration – Health care management	29 years at an operational level in EHS, and 3 years as a manager.	Coordination of the delivery of EHS

### 3.2 Roles and responsibilities in environmental health data management

With respect to the roles and responsibilities of managers in environmental health data management, the participants expressed their duties in different terms, which were related and supported one another. It was clear that they understood what is expected from them in the coordination of EHS, as well as the administration of information systems and quality assurance. The participants (*n* = 7; 70%) responsible for the coordination of EHS indicated that their duties include supervision of the work carried out by EHPs and derived reports, record keeping, data verification and validation, and timeous onwards submission of data to the DHIS. Data management support for subordinates was also mentioned, to streamline operational processes, and address emerging challenges.

Data management support was also mentioned to be inclusive of capacity-building, performance management and guidance on the implementation of policies, guidelines and standard operating procedures. Hence, participant #9 mentioned:

“Support and technical guidance on implementing the national guidelines and District Health Information Management System Policy as well as related Standard Operating Procedures… Coordination of programme performance reviews based on data. Provision of training on indicators, as well as on Web-DHIS.” (Participant #9)

Unanimity was observed from the managers again on the role that they play in planning, monitoring and evaluation within their settings and ensuring that the collected data get used to inform interventions.

The use of data for planning purposes and decision-making in the provision of EHS was also supported by the three (30%) managers responsible for monitoring and evaluation, informatics and quality assurance. One manager responsible for monitoring and evaluation said:

“My role as the Manager for Environmental Health Monitoring and Evaluation is multifaceted, encompassing the oversight of data collection, management and analysis processes, stakeholders’ engagement, policy support and capacity building to ensure the health and well-being of the community through effective use of environmental health information” (Participant #5)

These managers providing auxiliary support also highlighted their function of managing data repositories, including internal information systems, as well as providing support to those in front-line delivery of EHS, operational data matters and strategically:

“Provision of support to EHPs on the use of the internal Environmental Health Information Management System. Quality assurance audit… Utilisation of data from the system to serve as evidence for the implementation of operational and strategic plans.” (Participant #8)

### 3.3 Data quality improvement activities

Extending from the roles and responsibilities, the participants were asked to outline activities they engaged in to ensure that the data in their areas of responsibility were of good quality. All the participants (*n* = 10; 100%) mentioned data verification and validation against source documents (operationally and through data quality audits), capacity building, performance monitoring, standardisation of procedures and tools, data sharing, data feedback and discussions, and the safekeeping of data.

These activities were unanimous among the participants, even though they were reported as being practiced through different strategies. These implementation strategies included manual data quality checks, monitoring adherence to standard operating procedures on data management, discussions on data quality and verification during meetings, collaborative peer-reviews, as well as electronic data verification, quality checks and data cleaning on Web-DHIS before final submission.

As part of internal systems and strategies, one participant said:

“We have created systems which talk to weekly plans, weekly reports which then feed to monthly reports. To ensure that there is data validation and verification… Ensuring the submission of evidence in the form of checklists or reports on the work that has been covered, to ensure that the evidence speaks to the captured data. With all inspections conducted, checklists must be provided as evidence. To promote data quality, in terms of reliability. Putting internal control measures from time to time on working processes, to counter activities by EHPs as data collectors that may affect our data quality.” (Participant #10)

Participant #9, responsible for administrating the municipal information system and providing support to EHPs and managers responsible for the delivery of EHS mentioned the following activities:

“Ensuring that data collection tools are in sync with data demands or information needs. Ensuring that weekly and monthly data reports are completed in full and in alignment with the national indicators’ dataset and local environmental health data needs.”

In line with the above statement, another participant pointed his focus areas, which were key for data quality improvement:

“The activities I engage in to ensure data quality encompass various strategies aimed at enhancing the accuracy, reliability, completeness, and timeliness of the environmental health data we collect and use… Monitoring the performance of data collection and management activities against predefined indicators of data quality… and assessing the impact of interventions aimed at enhancing data quality”. (Participant #5)

These reported data quality improvement activities and implementation strategies were observed to be addressing data quality dimensions, as better information for utilisation requires data that is of good quality.

### 3.4 Use of environmental health data for planning and decision-making

In this context, the participants were asked to advise how environmental health data can be utilised during operational and strategic planning to inform decision-making. This was important to gain their perspectives and perceptions on leveraging the opportunities provided by the environmental health data, both operationally and strategically. The discussions on this theme were also premised on assessing the culture of data and information use among the participants.

The results revealed that environmental health data can be used to monitor the delivery of EHS against set norms and standards, address challenges and mobilise for resources. In the words of Participant #1, the above-mentioned point was articulated as follows:

“Environmental health data is used to gauge and foster compliance to National Norms and Standards for Environmental Health, especially with the frequency of inspections. To provide support to EHPs that get overwhelmed with the work, due to challenges and complexities in their areas. Data should be used in this regard to show a need for interventions. For mobilisation of working resources, and human resources… For financial support, to increase the capacity to deal with all environmental health issues and provide all services effectively and efficiently.”

Other participants (Participants #2 and #3) added to the above points by indicating that information derived from the data should be considered for effective planning and decision-making processes through identifying gaps and setting key performance areas. This was based on that:

“Environmental health data can play a crucial role in shaping operational and strategic plans. Operationally, it can help identify immediate health risks, guiding resource allocation for timely interventions. Strategically, trends in environmental data inform long-term planning, enabling proactive measures to address emerging public health challenges. Integrating these data into decision-making processes ensures a comprehensive approach to operational and strategic plans.” (Participant #6)

The submission by all the participants places data at the centre of risk assessments, allocation of resources, setting benchmarks and goals, as well as program design and implementation in terms of targeted interventions based on needs identified. To ensure continuous improvement in the provision of EHS, seven (70%) participants highlighted the role of data in performance monitoring and evaluation to identify areas of concern and drive the development of quality improvement plans. One of these participants is quoted below:

“Data that is on the system must be analysed to detect gaps and areas that need special interventions. The data must be used to plan, and cover identified gaps, as part of continuous improvement. Data must be used to adjust performance targets where necessary to make them realistic and reasonable.” (Participant #8)

The results show that all the participants had a positive perception of data use and saw its value in terms of providing evidence for decision-making: Participant #9 said:

“Environmental health data need to assist in identifying areas that have red flags in terms of environmental factors that threaten human health, to receive necessary interventions. Conduct trend analysis and patterns using data. Conduct data profiling, especially on issues like complaints and other miscellaneous issues.”

These results revealed managers' positive attitudes, culture and zeal in utilising environmental health data to improve service delivery and make an impact in the community, enabling better health outcomes.

### 3.5 Opportunities of effective environmental health data utilisation

Continuing from the above section, several opportunities from the effective utilisation of environmental health data emerged. This showed the power of data in the provision of EHS, and the significance of streamlined data management systems. Participant #5 outlined the following opportunities:

“(1) Risk management and emergency preparedness: Leverage historical data and trend analysis to predict potential environmental health risks and prepare for emergencies, such as epidemic outbreaks, natural disasters, or industrial accidents. (2) Stakeholder engagement and advocacy: Use environmental health data to engage stakeholders, including government officials, community leaders, and the public, in environmental health issues. Presenting data-driven evidence can be a powerful tool in advocating for policy changes, securing funding, and raising awareness about environmental health priorities. (3) Intersectoral collaboration: Analyse environmental health data in the context of broader social determinants of health, such as housing, education, and urban planning. This facilitates collaboration with other sectors, ensuring that operational and strategic plans are comprehensive and address the root causes of environmental health issues. (4) Policy development: Utilise environmental health data utilised to inform the development of local, regional, and national policies that protect public health and the environment. Data can provide the evidence base needed to justify new regulations, standards, and guidelines.”

From the above excerpt, as well as submissions by the other nine (90%) participants, it was clear that all the participants understood the value of data in depicting a state of environmental health conditions and issues for better planning and deployment of interventions, as well as risk communication. The responses to the theme of data utilisation outlined how the managers understood the use of data in their settings, as well as associated opportunities and increased outputs that can be leveraged to create more value and achieve greater efficiency.

### 3.6 Challenges in environmental health data management and utilisation

This theme outlined the challenges that the participants reported to experience in collecting, collating, storing, analysing, reporting and using environmental health data and information. Some of the key challenges that emerged were interrelated, from non-prioritisation of EHS to a lack of funding, a shortage of staff, the unavailability of technical tools for trade and smart devices to collect data in real time, and non-modernisation of environmental health data management systems. These challenges are categorised into the sub-themes below.

#### 3.6.1 Lack of funding

The lack of funding was outlined as a challenge by eight (80%) participants. Participant #1 mentioned that:

“Challenges include the availability of very little funds on the budget of the municipality, to provide EHS”.

This as a result also affects environmental health data collection, analysis, interpretation, dissemination and utilisation. Participant #8 indicated that:

“The lack of funds to provide hand-held mobile devices to be used to conduct inspections and environmental health investigations is also a challenge.”

Other participants raised that, the whole digital transformation journey to automate manual processes and improve operational efficiencies is affected by the lack of funds. In the words of Participant #5, the following response was mentioned:

“Securing the necessary funding for digital transformation projects, including the purchase of hardware, software, and training programs, is a significant challenge.”

Participants #2, #3 and #7 also mentioned that the unavailability of funds affect the modernisation of working systems, lead to shortage of staff, and insufficient tools of the trade. This also affects environmental health data management and information utilisation.

#### 3.6.2 Non-prioritisation of environmental health services

The non-prioritisation of EHS in the municipalities, in sync with the lack of funding emerged as part of the challenges, reported by 5 (50%) participants. Participant #1 said:

“The Department is understaffed… Environmental Health is not seen as a core function of municipalities, whereas it is core and essential. This disregard for Environmental Health led to this Department being least prioritised in the Municipality”.

The same sentiment was shared by Participant #10, who indicated that:

“EHS is not fully assimilated and integrated into the district municipalities after the services were transferred (devolution) from the Provincial Department of Health. Much work is needed for the promotion and prioritisation of EHS”.

#### 3.6.3 Poor coordination and centralisation of environmental health data

Poor coordination and centralisation of data also emerged from the results, as it was found that there are datasets that do not get reported or utilised for planning and decision-making. Participant #1 indicated that:

“Environmental Health is not reporting on all the work that we do. But we are guided by National Indicators Dataset and Provincial Indicators Dataset through the DHIS. Environmental Health goes beyond what is listed in the Scope of Practice and Job Descriptions, but we are not reporting that work to structures and other key stakeholders.”

The same sentiment was shared by another participant, who indicated that the environmental health data reported to the DHIS lacked granularity, for practitioners, managers and senior management to analyse and inform their decisions:

“Most challenges are related to the senior management that is unable to analyse and inform the EHS operations” (Participant #4)

Expanding from the above, a participant, from a monitoring and evaluation point of view, indicated that most challenges are systems-based in terms of environmental health data management and utilisation. Requiring adequate infrastructure to streamline data management and utilisation through digital technologies. These were the challenges outlined by Participant #5:

“(1) Inconsistent data collection methods in different offices… making it difficult to aggregate and analyse accurately (2) Limited access to or knowledge of advanced analytical software and tools restricts our ability to perform in-depth analyses. (3) Producing timely reports that can inform immediate action is difficult, especially when dealing with large and complex data sets. (4) Resistance from staff or stakeholders who are accustomed to traditional methods of operation can hinder the adoption of digital technologies.”

#### 3.6.4 Data quality, storage and interoperability challenges

Owing to mainly paper-based existing systems in the municipalities, other challenges that were expressed included data quality issues, as well as lack of interoperability to enable data sharing and integration. Participant #6 mentioned that:

“Ensuring accuracy and reliability of collected data can be challenging due to varying data sources and collection methods… Integrating diverse data formats and sources into a cohesive system for collation and analysis poses interoperability challenges.”

Ensuring proper storage of records, data security and privacy was mentioned as a challenge as well. Participant #5 cited that:

“Protecting sensitive health information from unauthorized access or breaches is a critical concern, especially in this digital age”.

Participant #8, from one of the municipalities that reported having an internal digital information system, said:

“The current challenge is the access to old records/files of premises, as the file cabinets that were used before the introduction of the new system collapsed and interrupted the filing plan. Therefore, the files storage room is a mess, that requires a special intervention for archiving, and scanning all these documents or records into the new system for onward storage”.

Participant #9 mentioned that:

“Currently records of data are stored in paper-based source documents, and data is captured in computers and USBs. Which makes them vulnerable to being lost or destroyed”.

Participant #10 also alluded that in their municipality:

“Data storage is an issue, as we do not have a filing system and a designated storage”.

### 3.7 Prospective solutions to improve environmental health data management and utilisation

To pave the way forward in environmental health data management and utilisation, the participants provided solutions that they deemed as required at this juncture. This is where suggestions on the digitalisation and procurement of digital tools arose. The issue of funding for digitalisation emerged as vital for improving data management, unlocking streamlined operational efficiencies and increasing decision-making capabilities to improve service delivery:

“It will not be difficult to digitise the current systems, once funding and technological support is provided.” (Participant #1)

“…move away from paper-based reliance systems and adopt artificial intelligent systems to red flag potential threats and generate reports and models for solutions.” (Participant #6)

“We need hand-held devices or portable gadgets like tablets. Which must be designed in such a way that they can store checklists. These devices are to be used to conduct inspections, signing digitally and sending reports digitally while in the field.” (Participant #10)

It was clear from the participants that there is a need to review and modernise the existing paper-based systems, as well as enrolling change management and capacity-building to leave no datasets and no one behind, in the transition. This was considered important for embracing change and its challenges while addressing knowledge, attitudes, perceptions and practices:

“The rapid pace of technological advancement means that institutions must continuously invest in training and upgrading systems to stay current.” (Participant #5)

“The training of EHPs must be continuous when a digital system is introduced to leave no one and data behind.” (Participant #7)

One participant noted that the country needs to have a single digital system, as a central source of environmental health data, for secure storage and record keeping, generation of reports in real-time, spatial visualisation of data, and application of data analytics. A shared view among the participants was that the use of a digital system and smart devices can reduce the administrative burden on environmental health professionals, increase work output and enable localised evidence-based interventions:

“It is important to add geographic information systems functionality on our digital system, to enable spatial visualisation of data. The country must implement one uniform digital environmental health information system at a national scale so that municipalities won't have many different systems… This will help to ensure that there is access to view the environmental health status in the country, province or municipality by just a click of a button and be able to generate any reports.” (Participant #8)

“The use of digital tools can also reduce the time that EHPs spend in offices for administration, which can be automated… The COVID-19 Pandemic showed us that we can work remotely, but we need the tools. The digital tools should also assist in terms of increasing our work output.” (Participant #10)

The above results in this theme demand that immediately, municipalities must maximise available technologies in their settings to kickstart the digital transition, implement continuous capacity-building programs and instil the culture of data utilisation. In the medium term, funding must be secured for resources and the digital infrastructure to be set up. In the long-term, the enrolment of a national digital environmental health information system and the adoption of artificial intelligence emerged as part of the key solutions.

## 4 Discussion

The ability to harness data effectively and make informed decisions is a defining factor for prosperity in improving the provision of services. Data are central to planning and decision-making to guide strategies, choices and actions. In this study, all the participants understood and valued the importance of data in this regard, as well as their roles and responsibilities. Clear roles and responsibilities are vital for the successful implementation of a routine health information system (23). Their definition and understanding among different role players facilitate improved data management, sharing and utilisation (24). The results of this study revealed the importance of auxiliary support sections in the rendering of EHS in terms of monitoring and evaluation, quality control and data management. However, nothing appeared from the results on the use of data for research as a responsibility for the interviewed managers, indicating neglect. Other governmental agencies, as well as academia and research institutes, non-governmental organisations, and the private sector all require health-related data to play a role in their settings to strengthen the health system (25, 26). Less was spoken as well about data feedback, which assists in discussions to address challenges and non-compliances, optimise best practices and enable continuous improvement in terms of data quality, and adherence to set standard guidelines. In a study that was conducted in Ethiopia, it was observed that health practitioners who received regular feedback were two times more interested in using their routine health information system compared to health practitioners who did not receive any feedback (27). Therefore, the less use of data feedback in this study, indicate that associated opportunities were missed by the managers, to, inter alia, provide support, address change management and improve data quality in their settings.

Data quality is one of the important factors in the successful implementation of a routine health information system, as it is key in the generation of reliable information (28). This was emphasised by the results of this study in terms of the activities that the participants engaged in to improve the quality of their environmental health data. From data verification and validation to data quality checks, capacity building, adherence to guidelines, safekeeping of data, data sharing and data feedback. These activities were outlined amid various reported challenges in terms of the lack of automated systems and tools for trade. The same applied when data were used for planning and decision-making. It is clear from the results that the interviewed managers understood the power of data in improving the provision of EHS, going beyond satisfying reporting obligations but rather to serve for decision support. In a study by Nicol et al. (29) on perceptions of data-informed decisions, managers’ inability to use information suggested that decisions on the provision of services were not always made on the basis of evidence from data, which detrimentally influenced the delivery of health services. The use of data is vital in enabling health institutions to be well managed and deliver the best outcomes (30). The results of this study indicated that this can be achieved through placing data at the centre of institutional governance, risk assessments, allocation of resources to areas where they are needed the most, policy and strategy development, stakeholder engagement, as well as performance monitoring and evaluation. As a result, it is evident that through data-driven insights, EHS delivery can be improved to be impactful in the community in terms of enabling better health outcomes. The managers who participated in this study showed a common interest in the culture of data-driven decision-making. Involved management and leadership are deemed key drivers of prosperity in data management and broader successful implementation of routine health information systems (31, 32). Hence, managers should lead, by example, in advancing transformation, as well as in sharing values and instilling a culture of data use (11). In addition, at all levels of the health system, managers should lead the use of data for objective and scientific decision-making, programme development and budgeting, as well as providing support to improve the health system (33).

In the journey of effectively utilising data for improved service delivery, various barriers and challenges exist, mostly in low- to middle-income countries (34). In this study, the lack of support, inadequate funding, limited resources, poor data coordination and centralisation, non-modernisation of systems, fragmentation of reporting systems, and lack of interoperability limited the potential for effective and efficient management and utilisation of environmental health data. This finding is consistent with the findings of Barron et al. (33), who reported that South Africa's DHIS collects extensive health and health system data, but its current use for decision-making is often limited by its lack of integration and timeous production, which is due mainly to data management practices. Paper-based systems have bureaucratic delays that affect data timeliness, availability, accessibility and quick utilisation (35). Hence, in this study, suggestions such as the centralisation of environmental health data management through a country-wide digital integrated environmental health information system and the procurement of smart hand-held devices and other technical tools for EHPs emerged to review and modernise the existing paper-based systems. This is in line with the recommendations that were provided by Wright and Street, on their paper on why big data can help to solve environmental health ills in South Africa (7). Other suggestions that emerged from this study, include applying change management in the environmental health community, enrolling in capacity building, strengthening quality assurance, and exploring emerging technologies for environmental health intelligence.

This study was able to identify weaknesses and threats that need to be attended to, as well as strengths and opportunities that should be leveraged to improve the management and utilisation of environmental health data. In order to promote safe and healthy environments where people live, work, learn, and play, the significance of data has also been shown when it comes to the provision of EHS. The suggested modernisation and improved environmental health data management in this study corroborates with the findings of a study that was conducted in the City of Johannesburg, South Africa, which also called for reforms in data management to change the way EHPs work, enhance record-keeping, increase efficiency, and promote data-driven decision-making (17). Therefore, this study reflects the dire need of strengthening EHS in the country, through improved data management. The participants that formed part of the study were experienced and knowledgeable on environmental health data management, which formed part of their responsibilities to different extents. The above are the strengths of this study.

In terms of its limitations, due to the lack of funding and the vastness of South Africa, this study targeted municipalities that provided EHS in the KwaZulu-Natal province. Furthermore, data was not collected from all these municipalities in this province, as managers from six municipalities were interviewed. Managers from the other four municipalities declined to participate, and two municipalities didn't agree to form part of the study. All the managers that were interviewed were environmental health professionals, indicating that other professionals (i.e., nurses, medical practitioners, administrative clerks, environmental management scientists, and others) that might be involved, to a certain degree, in the generation, management, and use of environmental health-related data were excluded. For future research, a country-wide status quo assessment of environmental health data management in South Africa is recommended. Municipalities with digital environmental health information systems also need to be studied, to allow best practices and systems to be shared, as part of the groundwork for a national integrated digital environmental health information system.

## 5 Conclusion

This study emphasises the central role and importance of managers in data management and information utilisation to improve service delivery and enable better health outcomes in the community. However, there are challenges that require a holistic approach in the health system to review and modernise the current systems for data management and access to technological infrastructure and tools. This review and modernisation are needed to match managers' enthusiasm and motivation in leveraging the power of data to drive effective and efficient EHS. In that paradigm shift, managers have a duty to drive transformation and instil a culture of data use in their institutions, as leaders, visionaries, change agents and advocates for impactful service delivery.

## Data Availability

The raw data supporting the conclusions of this article will be made available by the authors, without undue reservation.
